# Association between Circulating Growth Differentiation Factor 15 and Cirrhotic Primary Biliary Cholangitis

**DOI:** 10.1155/2020/5162541

**Published:** 2020-10-29

**Authors:** Zhanyi Li, Yu Liu, Xiangyong Li, Yuankai Wu, Fangji Yang, Qiwan Mo, Yutian Chong

**Affiliations:** ^1^Department of Infectious Diseases, Third Affiliated Hospital of Sun Yat-sen University, Guangzhou, Guangdong 510630, China; ^2^Department of General Surgery (Thyroid and Breast), Third Affiliated Hospital of Sun Yat-sen University, Guangzhou, Guangdong 510630, China; ^3^Physical Examination Center, Third Affiliated Hospital of Sun Yat-sen University, Guangzhou, Guangdong 510630, China

## Abstract

Primary biliary cholangitis (PBC) is a common condition that usually shows a progressive course towards cirrhosis without adequate treatment. Growth differentiation factor 15 (GDF15) plays multiple roles in various pathological conditions. The overall role of circulating GDF15 in cirrhotic PBC requires further investigation. Twenty patients with cirrhotic PBC, 26 with non-cirrhotic PBC, and 10 healthy subjects were enrolled between 2014 and 2018, and the serum levels of GDF15 were measured via enzyme immunoassay. The correlations between serum GDF15, weight, biochemical parameters, and the prognosis were analysed. Serum levels of GDF15 were significantly higher in cirrhotic PBC patients than in non-cirrhotic PBC patients or healthy controls (*p* = 0.009 and *p* < 0.001, respectively). The circulating GDF15 levels strongly correlated with weight changes (*r* = −0.541, *p* = 0.0138), albumin (*r* = −0.775, *p* < 0.0001), direct bilirubin (*r* = −0.786, *p* < 0.0001), total bile acids (*r* = 0.585, *p* = 0.007), and C-reactive protein (*r* = 0.718, *p* = 0.0005). Moreover, circulating GDF15 levels strongly correlated with the Mayo risk score (*r* = 0.685, *p* = 0.0009) and Model for End-stage Liver Disease score (*r* = 0.687, *p* = 0.0008). Determined by the area under the receiver operating characteristic curves, the overall diagnostic accuracies of GDF15 were as follows: cirrhosis = 0.725 (>3646.55 pg/mL, sensitivity: 70.0%, specificity: 69.2%), decompensated cirrhosis = 0.956 (>4073.30 pg/mL, sensitivity: 84.62%, specificity: 100%), and cirrhotic biochemical non-responders = 0.835 (>3479.20 pg/mL, sensitivity: 71.43%, specificity: 92.31%). GDF15 may be a useful and integrated biochemical marker to evaluate not only the disease severity and prognosis but also the nutrition and response to treatment of cirrhotic PBC patients, and its overall performance is satisfactory. Therapy targeting GDF15 is likely to benefit cirrhotic PBC patients and is worth further research.

## 1. Introduction

Primary biliary cholangitis (PBC) is an immune-mediated inflammatory cholestatic liver disease characterized by nonsuppurative destructive cholangitis and interlobular bile duct destruction. It is a chronic progressive condition leading to end-stage liver disease including liver cirrhosis (LC) and hepatocellular carcinoma and their associated complications that commonly require liver transplantation [1–3]. It has been reported that without effective therapy, the median time of progression to extensive liver fibrosis is 2 years with about one-third of the patients remaining in early-stage disease over a follow-up period of 4 years [4–6]. Conversely, several early-stage studies have demonstrated that the incidence of progression to LC after 6 years of follow-up was 1 in 2 for patients who received penicillamine or placebo (compared to 1 in 10 for patients who received ursodeoxycholic acid) [7]. Cirrhosis is a great burden on public health care. In 2010, it was the twelfth leading cause of mortality worldwide, responsible for approximately 1 million deaths [8]. Vibration-controlled transient elastography (VCTE) and liver biopsy were commonly used to diagnose cirrhosis. The VCTE is recommended as the initial assessment for significant liver fibrosis and cirrhosis, and it is a quick, portable point-of-care test. But the reliability of VCTE may be influenced by operator experience, obesity, ascites, narrow intercostal spaces, hepatic inflammation, cholestasis, and hepatic congestion. It is only a surrogate marker for the diagnosis [9]. Liver biopsy can diagnose the cirrhosis accurately but is an invasive method and not feasible in all patients and can pose complications of pain, haemorrhage, infection, perforation of a neighbouring organ, or even death. Moreover, small specimen's size, sampling error, and variability with inter- and intraobserver reliability may lead to poor reproducibility for liver biopsies. These disadvantages limit its broad application in cirrhosis diagnosis [9]. Early serum biomarker screening in patients at a high risk of developing LC may reduce the morbidity and mortality rates and decrease medical costs. However, the sensitivity and specificity of currently available serum biomarker for cirrhosis diagnosis are unsatisfactory. Optimal diagnostic serum biomarkers for cirrhosis are needed.

Growth differentiation factor 15 (GDF15), also known as macrophage inhibitory cytokine 1 (MIC-1), is a stress-responsive cytokine belonging to the transforming growth factor beta (TGF-*β*) superfamily which includes several proteins involved in tissue homeostasis, differentiation, remodelling, and repair [10, 11]. GDF15 has been demonstrated to play multiple roles in various pathological conditions such as cancer, inflammatory diseases, cardiovascular diseases, lung diseases, kidney injury, and metabolic disorders [12–16]. Recent studies have found that GDF15 can induce anorexia and fat and lean body mass loss [17, 18], and there appears to be a consistent correlation between an increase in the serum levels of GDF15 and a decrease in the markers of nutrition [19]. In addition, it was reported that an elevated serum GDF15 level is detected during hepatitis C virus infection, which is potentially caused by either viral agents or host stress/injury, or by both. GDF15 may contribute to HCV pathogenesis by altering the signalling and growth of host and represents a potential diagnostic serum biomarker and interventional target for viral hepatitis [10]. Measuring serum levels of GDF15 is a noninvasive and simple-to-use test. However, the clinical relevance of the relationship between circulating GDF15 and end-stage liver diseases, such as in PBC patients with cirrhosis, has not been reported.

The aim of the present study was to measure the serum levels of GDF15 in cirrhotic PBC patients and examine the relationship between serum GDF15 and changes in the body weight and clinical parameters to determine the role of GDF15 in cirrhotic PBC patients. Illustrating the biological function of circulating GDF15 in cirrhosis will help promote its potential application in the diagnosis and targeted therapy of cirrhotic PBC patients.

## 2. Materials and Methods

### 2.1. Patients

All enrolled patients were diagnosed and followed up at the Third Affiliated Hospital of Sun Yat-Sen University between 2014 and 2018. The diagnosis of PBC was based on the American Association for the Study of Liver Diseases (AASLD) Practice Guidelines [20]. Cirrhosis was diagnosed using either an imaging technique such as ultrasonography, magnetic resonance imaging, or computed tomography or via liver biopsy. PBC patients received ursodeoxycholic acid (UDCA) at a standard dose of 13-15 mg/kg daily. Decompensated cirrhosis was defined based on the complications that the patient had such as variceal bleeding, ascites, encephalopathy, and jaundice [21]. Ten individuals with no abnormal clinical (according to previous medical records), physical, or biochemical findings were included as healthy controls in this research. The standard clinical laboratory methods were used to measure the biochemical parameters. The “Paris criteria” were employed to define the biochemical response to UDCA treatment [22], and the Model for End-stage Liver Disease (MELD) score [23] and Mayo risk score (MRS) [24] were employed to evaluate the prognosis of the patients.

Patients' body weights were measured on admission to the hospital and compared with the body weights described in their previous medical records. Malnutrition was evaluated according to the Global Leadership Initiative on Malnutrition (GLIM) criteria for the diagnosis of malnutrition [25].

The exclusion criteria were applicable to patients who had any of the following: (a) heart failure, renal disease, or pulmonary disease; (b) other hepatological pathologies such as viral hepatitis, primary sclerosing cholangitis, alcoholic liver disease, and fatty liver disease; (c) any carcinoma; and (d) long-term usage of diuretics for ascites or oedema before admission to the hospital.

The study protocol was approved by the ethics committee of the Third Affiliated Hospital of Sun Yat-sen University. Blood samples were acquired after obtaining written consent from the patients.

### 2.2. Measurement of Serum GDF15 Levels

We used enzyme-linked immunosorbent assay (ELISA) (Abcam, UK) to measure the levels of serum GDF15 as per the manufacturer's instructions for all patients at the time of hospital admission.

### 2.3. Statistical Analysis

The baseline demographic and clinical characteristics are listed as means with standard error of the mean (SEM) or percentage. Student's *t* test or the Mann–Whitney *U* test was employed to estimate continuous data whereas the chi-square test or Fisher's exact test was employed to estimate categorical data. Pearson's correlation coefficient or Spearman's rank correlation was employed to assess the correlation of data. All parameters exhibiting strong correlations in the univariate analysis as covariates were subjected to multiple linear regression. Multiple linear regression analysis was conducted to detect independent relationships and adjust the effects of covariates. Receiver operating characteristic (ROC) curves were used to compare the diagnostic values of GDF15. The areas under the curves were calculated by selecting clinically relevant threshold levels to optimize the sensitivity and specificity. SPSS version 19 (IBM, Armonk, NY, USA) was used for the statistical analyses. All analyses were two-sided, and differences were defined as statistically significant when *p* < 0.05.

## 3. Results

### 3.1. Demographic and Clinical Characteristics of Patients with PBC and Healthy Controls

Forty-six PBC patients (26 without cirrhosis and 20 with cirrhosis) and 10 healthy controls were included in this study (see Table 1). The age was not different in non-cirrhotic and cirrhotic PBC patients and healthy controls (52.12 ± 2.11 vs. 53.95 ± 2.63 vs. 53.20 ± 4.13 years, *p* > 0.05). Serum liver enzyme (alanine transaminase (ALT), aspartate transaminase (AST), alkaline phosphatase (ALP), and gamma-glutamyl transpeptidase (GGT)) levels, bilirubin (total bilirubin (TBIL) and direct bilirubin (DBIL)) levels, and total bile acid (TBA) levels were significantly higher in both non-cirrhotic and cirrhotic PBC patients than in healthy controls. The international normalized ratio (INR) was significantly higher in patients with PBC (both non-cirrhotic and cirrhotic) than in healthy controls. In contrast, serum albumin (ALB) levels were significantly lower in PBC patients regardless of cirrhosis than in healthy controls. In addition, the TBA, INR, MELD score, and Mayo risk score of cirrhotic PBC patients were significantly higher than those of non-cirrhotic PBC patients. Serum levels of C-reactive protein (CRP) were significantly higher in cirrhotic PBC patients than in healthy controls (see Table 1).

The weight changes were 0.300 ± 0.44 kg (0.47% ± 0.71%) in healthy controls, −0.962 ± 0.51 kg (−1.49% ± 0.77%) in non-cirrhotic PBC patients, and −1.50 ± 0.43 kg (−2.71% ± 0.74%) in cirrhotic PBC patients. Weight loss was significantly higher in cirrhotic PBC patients than in non-cirrhotic PBC patients (*p* = 0.015) and healthy control individuals (*p* = 0.011). The BMI was significantly lower in cirrhotic PBC patients than in non-cirrhotic PBC patients (19.82 ± 2.12 vs. 21.39 ± 2.09 kg/m^2^, *p* = 0.016) and healthy controls (19.82 ± 2.12 vs. 23.27 ± 2.51 kg/m^2^, *p* < 0.001). The incidence of malnutrition was 45% (9/20) in cirrhotic PBC patients and 15.38% (4/26) in non-cirrhotic PBC patients. None of the healthy controls presented with malnutrition. The incidence of malnutrition was higher in cirrhotic PBC patients than in non-cirrhotic PBC patients (*p* = 0.046) and healthy controls (*p* = 0.013).

### 3.2. Serum Levels of GDF15 in Patients with PBC (Cirrhotic and Non-cirrhotic) and Healthy Controls

**Figure 1:**
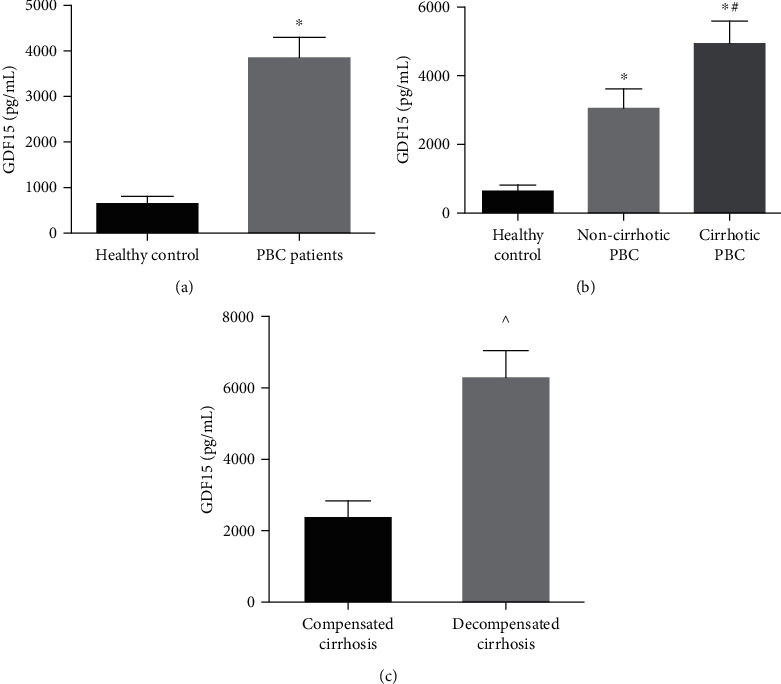
Serum GDF15 concentrations in PBC patients with and without cirrhosis and healthy controls.

Serum levels of GDF15 were significantly higher in PBC patients than in healthy controls (3858.73 ± 449.19 vs. 656.58 ± 146.13 pg/mL; *p* < 0.001) (see Figure 1(a)). Serum levels of GDF15 were significantly higher in non-cirrhotic PBC patients than in healthy controls (3037.41 ± 568.91 vs. 656.58 ± 146.13 pg/mL; *p* = 0.002) (see Figure 1(b)), and serum levels of GDF15 were significantly higher in cirrhotic PBC patients than in healthy controls (4926.44 ± 662.84 vs. 656.58 ± 146.13 pg/mL; *p* < 0.001) or non-cirrhotic PBC patients (4926.44 ± 662.84 vs. 3037.41 ± 568.91 pg/mL; *p* = 0.009) (see Figure 1(b)). Moreover, serum levels of GDF15 were significantly higher in PBC patients with decompensated cirrhosis than in PBC patients with compensated cirrhosis (6679.31 ± 828.27 vs. 2784.04 ± 477.06 pg/mL; *p* < 0.001) (see Figure 1(c)).

### 3.3. Clinical and Laboratory Parameters Related to GDF15 in Cirrhotic PBC Patients

**Figure 2:**
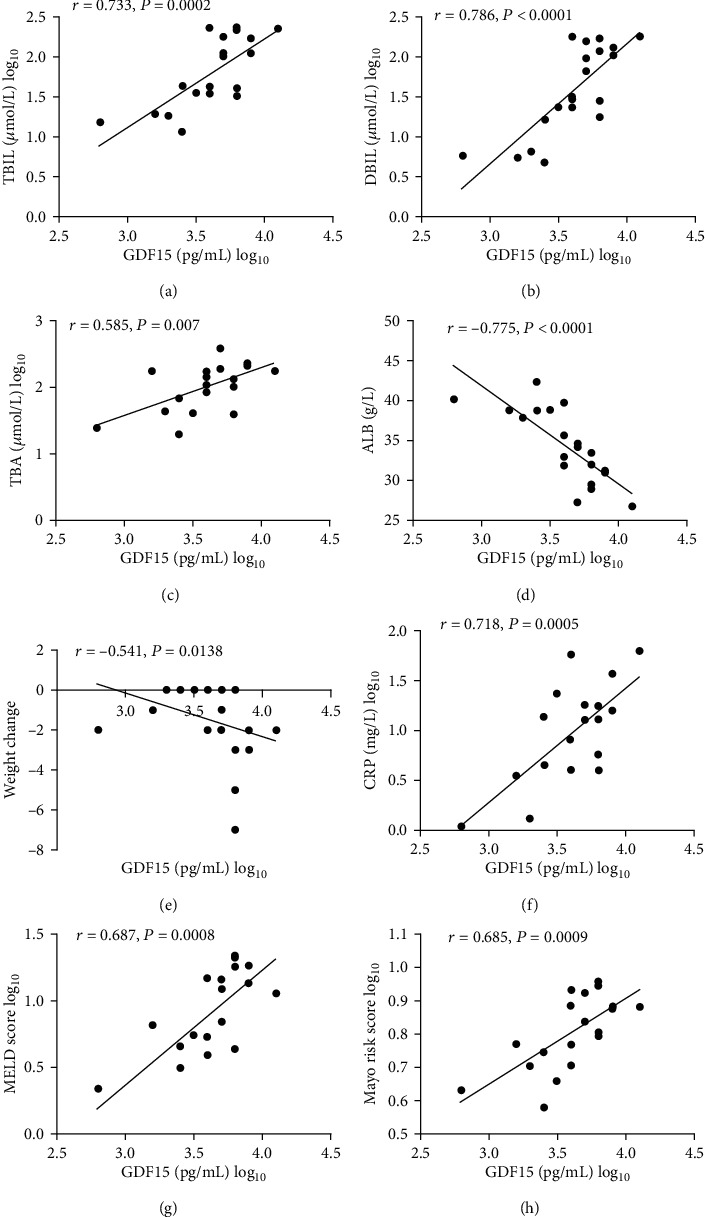
Laboratory and clinical parameters associated with GDF15 in cirrhotic PBC patients (*μ*mol/L).

Serum bilirubin (TBIL, DBIL) and TBA levels are typical markers of cholestasis. Prominently, positive correlations were detected between serum GDF15 levels and TBIL (*r* = 0.733, *p* = 0.0002), GDF15 and DBIL (*r* = 0.786, *p* < 0.0001), and GDF15 and TBA (*r* = 0.585, *p* = 0.007) in cirrhotic PBC patients (see Figures 2(a)–2(c)). There was a negative correlation between ALB and GDF15 (*r* = −0.775, *p* < 0.0001) in cirrhotic PBC patients (see Figure 2(d)). We also detected a negative correlation between the serum levels of GDF15 and weight changes (*r* = −0.541, *p* = 0.0138) in cirrhotic PBC patients (see Figure 2(e)). Serum levels of CRP, which is an acute-phase protein expressed in the liver, rise in response to inflammation. CRP is a typical marker of the response to inflammation. A positive correlation was detected between serum GDF15 levels and CRP (*r* = 0.718, *p* = 0.0005) (see Figure 2(f)).

The MELD score (based on a calculation including the INR and bilirubin and creatinine levels) is commonly employed to assess disease severity and outcomes in patients with liver diseases [21]. In the present study, GDF15 levels strongly correlated with the MELD score (*r* = 0.687, *p* = 0.0008) (see Figure 2(g)).

The Mayo risk score (based on a series of potential risk factors including age, albumin and bilirubin levels, prothrombin time, and the presence of peripheral oedema and diuretic treatment) is typically employed to assess the outcomes in PBC patients [22]. In the present study, GDF15 levels strongly correlated with the Mayo risk score (*r* = 0.685, *p* = 0.0009) (see Figure 2(h)).

**Figure 3:**
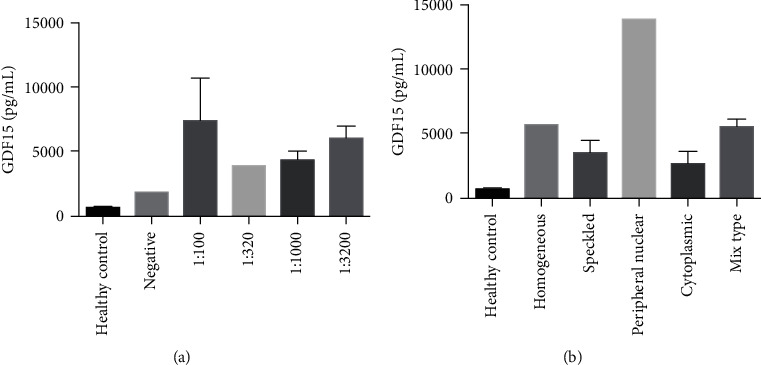
Serum GDF15 concentrations in cirrhotic PBC patients with different ANA titres and ANA patterns.

GDF15 levels did not differ significantly in cirrhotic PBC patients with different ANA titres (*p* = 1.000) and ANA patterns (*p* = 0.114) (see Figure 3).

Univariate regression analysis showed significant positive correlations between GDF15 and bilirubin (TBIL and DBIL) levels and GDF15 and CRP and TBA levels but significant negative correlations between GDF15 and ALB levels (see Table 2). We also detected a significant positive relationship between serum GDF15 levels and patients' MELD and Mayo risk scores (see Table 2). Multivariate analysis of these data revealed that ALB was an independent variable of serum GDF15 levels (*p* = 0.038) in cirrhotic PBC patients (see Table 3).

### 3.4. Serum GDF15 Levels, Patient Prognosis, and Biochemical Responsiveness in Cirrhotic PBC Patients

There were 7 biochemical responders and 13 biochemical non-responders among the 20 cirrhotic PBC patients. Prominently, serum levels of GDF15 were significantly higher in biochemical non-responders (patients that failed to respond to treatment) than in biochemical responders (5972.83 ± 809.45 vs. 2983.14 ± 757.12 pg/mL, *p* = 0.014) (Table 4). Serum bilirubin (TBIL and DBIL), INR, CRP, and the Mayo risk scores were significantly higher in biochemical non-responders than in biochemical responders. The clinical and laboratory features of biochemical responders and non-responders are presented in Table 4.

**Figure 4:**
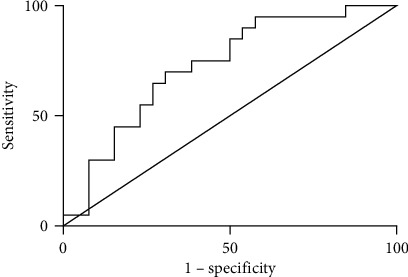
GDF15 ROC curve shows the comparison between cirrhotic PBC and non-cirrhotic PBC patients.

ROC curve analysis was used to define the optimal cut-off to determine the sensitivity and specificity of serum GDF15 for categorizing cirrhotic PBC patients versus non-cirrhotic PBC patients. The area under the ROC curve (AUROC) was 0.725 (95% CI 0.578-0.872), with a sensitivity of 70.0% (95% CI 0.457-0.881), specificity of 69.2% (95% CI 0.482-0.857), and an optimal cut-off value of 3646.55 pg/mL (see Figure 4). The results showed that the serum levels of GDF15 could be effectively used to differentiate cirrhotic patients from other patients in the cohort with PBC.

**Figure 5:**
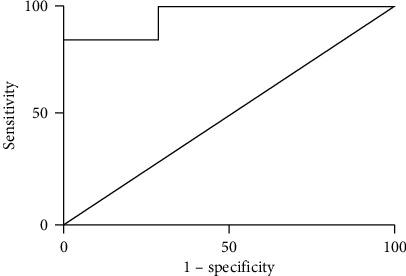
GDF15 ROC curve shows the comparison between PBC patients with decompensated cirrhosis and PBC patients with compensated cirrhosis.

ROC curve analysis was used to define the optimal cut-off to determine the sensitivity and specificity of serum GDF15 for categorizing PBC patients with decompensated cirrhosis versus PBC patients with compensated cirrhosis. The area under the ROC curve (AUROC) was 0.956 (95% CI 0.873-1.000), with a sensitivity of 84.62% (95% CI 0.546-0.981), specificity of 100% (95% CI 0.590-1.000), and an optimal cut-off value of 4073.30 pg/mL (see Figure 5). The results showed that the serum levels of GDF15 could be effectively used to differentiate patients with decompensated cirrhosis among cirrhotic PBC patients.

**Figure 6:**
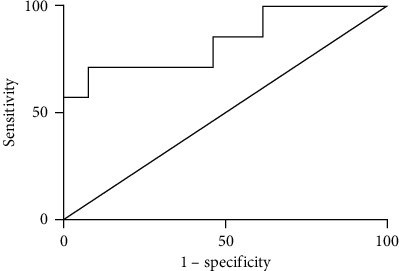
GDF15 ROC curve shows the comparison between biochemical responders and biochemical non-responders among cirrhotic PBC patients.

ROC curve analysis was used to define the optimal cut-off to determine the sensitivity and specificity of serum GDF15 for categorizing biochemical responders versus biochemical non-responders in cirrhotic PBC patients. The AUROC was 0.835 (95% CI 0.633-1.000) with a sensitivity of 71.43% (95% CI 0.290-0.963), specificity of 92.31% (95% CI 0.639-0.998), and an optimal cut-off value of 3479.20 pg/mL (see Figure 6). The results showed that the serum levels of GDF15 could be effectively used to differentiate biochemical non-responders among cirrhotic PBC patients.

## 4. Discussion

GDF15 is involved in the stress response program of different cell types after cellular injury and regulates inflammation [16, 26]. Previous studies have reported that GDF15 expression is rapidly induced following injury to hepatocytes and bile duct epithelial cells [16, 27]. In patients with PBC, bile duct lesions, biliary secretion impairment, and hepatocellular accumulation of toxic endogenous bile acids result in cellular damage and necroinflammatory lesions and fibrosis of the liver. The present study showed that the increase in the GDF15 levels was positively correlated with the degree of cholestasis and inflammation in cirrhotic PBC patients. The increase in GDF15 is most likely a response to cell stress/damage and inflammation caused by PBC. As it is a circulating cytokine, it is logical to hypothesize that GDF15 has a paracrine, autocrine, or endocrine action in PBC patients. Our findings are in accord with those of previous studies which suggested that serum GDF15 levels were elevated in patients with chronic liver diseases such as nonalcoholic fatty liver disease (NAFLD) and chronic hepatitis B or C virus infection [28, 29] and supplement those of previous studies. In addition, the present study also showed that serum levels of GDF15 were markedly increased in cirrhotic PBC patients, especially in decompensated cirrhotic PBC patients. Our findings are in accord with those of previous studies which suggested that the increase in GDF15 levels depends on the severity of fibrosis rather than hepatic cell injury/inflammation. Thus, GDF15 may be a good indicator of the severity of the liver fibrosis.

It has been reported that GDF15 is associated with multiple organ fibrosis such as atrial, renal, and pulmonary fibrosis [12–14]. Recently, the association between GDF15 and liver fibrosis has attracted increasing attention. Elevated GDF15 levels were found to be associated with advanced liver fibrosis in chronic liver diseases [28, 29]. Chronic and repetitive hepatocyte injury results in the overexpression of GDF15, and a dysregulation of GDF15 release may lead to prolonged stimulation of hepatic stellate cells (HSCs) and promote the progression of liver cirrhosis [28, 29]. GDF15 has been reported to not only directly stimulate transforming growth factor beta 1 (TGF-*β*1) expression [30] but also induce the phosphorylation of SMAD2 and SMAD3 proteins to activate human HSCs and induce fibrosis [31]. Serum GDF15 levels may be a potential biomarker of advanced fibrosis in chronic liver diseases.

The present study showed that serum GDF15 levels in cirrhotic PBC patients were markedly increased. Serum GDF15 levels of non-cirrhotic PBC patients were also moderately higher than those of healthy controls, but significantly lower than those of cirrhotic PBC patients. The ROC curve comparing cirrhotic PBC patients and non-cirrhotic PBC patients in the cohort suggested that GDF15 could differentiate LC with an AUROC of 0.725. These results demonstrated that GDF15 could serve as a serum biomarker of LC. Decompensated LC often has a high mortality rate, and it is essential to distinguish between compensated and decompensated cirrhosis when predicting patients' prognosis. Decompensated LC patients cannot tolerate the reliable but highly invasive diagnostic modality of liver biopsy. As to the noninvasive modality, current models including MELD scores cannot distinguish between compensated and decompensated cirrhosis and conventional radiological modalities for fibrosis assessment can only provide the morphological evaluation of liver fibrosis, so improved tools for early and noninvasive diagnosis of LC are urgently needed. The present study showed that serum GDF15 levels in PBC patients with decompensated cirrhosis were markedly increased. Serum GDF15 levels of PBC patients with compensated cirrhosis were also moderately higher than those of healthy controls, but significantly lower than those of PBC patients with decompensated cirrhosis. The ROC curve comparing PBC patients with decompensated cirrhosis and PBC patients with compensated cirrhosis in the cohort suggested that GDF15 could differentiate decompensated LC with an AUROC of 0.956. These results demonstrated that GDF15 could serve as a serum biomarker of decompensated LC.

Patients with cirrhosis are exceptionally vulnerable to developing malnutrition and some degree of cachexia because of the key role played by the liver in regulating the nutritional state and energy balance. It has been reported that the prevalence of malnutrition in LC ranges from 10% to 100% depending on the severity of the disease [32, 33]. In the present study, we found that there were negative correlations between ALB and GDF15 (*r* = −0.775, *p* < 0.0001) (see Figure 2(d)) and GDF15 and weight changes (*r* = −0.541, *p* = 0.0138) (see Figure 2(e)) in cirrhotic patients with PBC. Recently, the role of GDF15 in body weight regulation has been reported. In humans with chronic diseases and malignancies, GDF15 can suppress appetite and induce weight loss even in cachexia [34–36]. GDF15 may contribute to malnutrition in patients with cirrhotic PBC. The elevated circulating GDF15 levels in cirrhotic PBC patients may suppress appetite and reduce food intake, thus influencing nutrient intake. As a result, the synthesis of ALB and maintenance of body weight were influenced by serum GDF15 levels in this study.

Studies have shown that compared to the outcomes of well-nourished patients, malnourished patients with liver disease have poorer outcomes and higher morbidity rates due to major complications requiring hospitalization (71.3% vs. 38.2%, *p* = 0.002) as well as higher mortality rates (41.1% vs. 18.2%, *p* = 0.001) [37]. Early detection and treatment of malnutrition are imperative to improve patient outcomes [38, 39]. Identification of patients in the anorexia–cachexia spectrum who could gain clinical benefits from nutritional support and other therapies is a clinical problem that needs to be solved urgently. However, no well-validated biomarkers for predicting malnutrition and cachexia are available thus far. In the present study, we found that there were negative relationships between ALB and GDF15 (*r* = −0.775, *p* < 0.0001) (see Figure 2(d)) and GDF15 and weight changes (*r* = −0.541, *p* = 0.0138) (see Figure 2(e)) in cirrhotic PBC patients but a positive relationship between CRP and GDF15 (*r* = 0.718, *p* = 0.0005) (see Figure 2(f)). The ALB levels < 32 g/L and CRP levels > 5 mg/L form part of the diagnostic criteria for malnutrition and cachexia [40]. Thus, GDF15 may be useful as a biochemical marker to predict malnutrition and cachexia.

The primary characteristics of malnutrition and cachexia are inadequate nutrient intake, decreased or absent physical activity, and altered metabolism, partly due to a pathological systemic inflammatory response [41]. To improve malnutrition and cachexia, adequate nutrition should be provided to preserve and restore muscle mass and limit systemic inflammation [41]. Malnutrition and cachexia are the focus of many ongoing studies, but the optimum means for diagnosis or early detection have not been definitely identified thus far. It also appears that, in some patients, the dominant cause of weight loss, loss of muscle mass, and cachexia is a systemic inflammatory response, which emphasizes the importance of systemic inflammation as a target for therapeutic intervention [41]. Such targets should have the direct or indirect potential to stimulate anabolism and/or improve appetite [42]. GDF15 is involved in inflammation regulation [16, 26] and can suppress appetite [17, 18]. In murine models of tumours, mice overexpressing GDF15 showed weight loss, and the degree of weight loss was proportional to the elevation of serum levels of GDF15 [17]. This phenomenon of weight loss in murine models of tumours could be reversed by the utilization of monoclonal antibodies to GDF15 and reproduced by the utilization of recombinant GDF15 [17]. Moreover, it has been reported that serum levels of GDF15 might be associated with all-cause mortality in multiple diseases, and it is possible that disease-specific therapeutic interventions that decrease serum levels of GDF15 may also reduce the risk of mortality and increase longevity [43]. Treatment targeting GDF15 may improve symptoms as well as the nutrient status, thereby improving the outcome of cirrhotic PBC patients.

Recently, the role of serum GDF15 levels in predicting advanced liver fibrosis and severity of chronic liver disease in NAFLD, alcoholic liver diseases, and chronic hepatitis B and C was reported [28, 29]. However, there are limited data on the role of serum levels of GDF15 in patients with PBC. The MELD score is commonly employed to assess the disease severity and prognosis in patients with liver disease [23]. In the present study, a positive correlation was detected between serum levels of GDF15 and MELD scores (*r* = 0.687, *p* = 0.0008) (see Figure 2(g)) in cirrhotic PBC patients. This result demonstrated that serum levels of GDF15 reflect the disease state, and GDF15 could serve as a serum biomarker to indicate the severity of the disease in cirrhotic PBC patients.

The Mayo risk score is typically employed to assess the outcomes of PBC patients [24]. In the present study, a positive correlation was detected between serum levels of GDF15 and the Mayo risk score (*r* = 0.685, *p* = 0.0009) (see Figure 2(h)) in cirrhotic PBC patients. Assessment of the biochemical response indicated that serum GDF15 levels were significantly elevated in biochemical non-responders (see Table 4). The ROC curve comparing biochemical responders and non-responders in cirrhotic PBC patients suggested that GDF15 levels could be used to differentiate biochemical non-responders to UDCA treatment among cirrhotic PBC patients with an AUROC of 0.835. These results demonstrated that GDF15 could serve as a serum biomarker of treatment response to UDCA in cirrhotic PBC patients and potentially indicate the prognosis of cirrhotic PBC patients. This is a novel finding about the role of GDF15 in chronic liver diseases.

Despite these novel findings, this study has some limitations. Data regarding the muscle mass were lacking. This investigation was a small-scale single-centre cohort study and too limited in size to arrive at any definite conclusion. Large-scale multicentre cohort studies are needed to construct more accurate associations.

## 5. Conclusions

In conclusion, the present study is important as a supplement to previous studies on the role of GDF15 in chronic liver diseases [28, 29]. These findings provide evidence that GDF15 can predict liver fibrosis, severity response to UDCA treatment, and malnutrition in chronic liver disease. Measuring serum GDF15 levels, a noninvasive and simple-to-use test, could be potentially useful in evaluating the disease severity and prognosis of cirrhotic PBC patients, and it has its advantages over the existing prediction models. Serum levels of GDF15 have high sensitivity and specificity in differentiating between compensated and decompensated LC when compared to MELD score, and serum levels of GDF15 have high sensitivity and specificity in differentiating between cirrhotic PBC patients and non-cirrhotic PBC patients when compared to Mayo risk scores. In addition, it can predict malnutrition and cachexia that are associated with the disease severity and prognosis in cirrhotic PBC patients. No well-validated biomarkers for predicting malnutrition and cachexia in end-stage liver disease are available thus far, and improved tools for early and noninvasive diagnosis of LC are urgently needed. GDF15 may be a useful and integrated biochemical marker to evaluate not only the disease severity and prognosis but also the nutrition and response to treatment of patients with chronic liver diseases, and its overall performance is satisfactory. Therapy targeting GDF15 is likely to benefit cirrhotic PBC patients and is worth further research.

## Figures and Tables

**Table 1:** Clinical and laboratory parameters of PBC patients and healthy controls.

Feature	Healthy controls (*n* = 10)	Non-cirrhotic PBC patients (*n* = 26)	Cirrhotic PBC patients (*n* = 20)
Age (years)	53.20 ± 4.13	52.12 ± 2.11	53.95 ± 2.63
Gender (male)	4	3	7
Weight change (kg)	0.300 ± 0.44	−0.962 ± 0.51	−1.50 ± 0.43^∗^^#^
Weight change (%)	0.47% ± 0.71%	−1.49% ± 0.77%	−2.71% ± 0.74%^∗^^#^
>5% within past 6 months	0	2	4
BMI (kg/m^2^)	23.27 ± 2.51	21.39 ± 2.09^∗^	19.82 ± 2.12^∗^^#^
Low BMI (<18.5 if <70 years)	0	2	6
CRP (mg/L)	3.37 ± 0.49	8.89 ± 1.87	15.99 ± 1.75^∗^
CRP > 5 mg/L	0	13^∗^	12^∗^
Malnutrition (*n*, %)	0	4 (15.38%)	9 (45%)^∗^^#^
GDF15 (pg/mL)	656.58 ± 146.13	3037.41 ± 568.91^∗^	4926.44 ± 662.84^∗^^#^
ALT (U/L)	15.80 ± 1.98	113.00 ± 17.37^∗^	85.85 ± 15.27^∗^
AST (U/L)	21.80 ± 1.29	114.12 ± 15.64^∗^	102.10 ± 14.09^∗^
TBIL (*μ*mol/L)	8.56 ± 1.34	59.85 ± 12.49^∗^	95.69 ± 18.58^∗^
DBIL (*μ*mol/L)	2.91 ± 0.56	43.71 ± 10.78^∗^	69.87 ± 14.58^∗^
GGT (U/L)	25.80 ± 4.62	474.08 ± 84.86^∗^	309.60 ± 71.96^∗^
ALP (U/L)	60.30 ± 5.66	308.08 ± 41.13^∗^	269.95 ± 25.71^∗^
TBA (*μ*mol/L)	3.27 ± 0.34	81.18 ± 16.80^∗^	132.54 ± 19.57^∗^^#^
ALB (g/L)	43.98 ± 0.52	38.39 ± 0.85^∗^	34.24 ± 1.02^∗^^#^
GLB (g/L)	27.13 ± 1.16	34.75 ± 1.28^∗^	35.86 ± 2.25^∗^
INR	0.95 ± 0.01	1.04 ± 0.06^∗^	1.28 ± 0.09^∗^^#^
MELD score	N/A	5.73 ± 1.07	10.14 ± 1.47^#^
Mayo risk score	N/A	5.53 ± 0.29	6.58 ± 0.35^#^
ANA positive	0	25	19
Anti-SP100	0	1	1
Anti-GP210	0	1	4
AMA positive	0	22	16
AMA-M2 positive	0	9	6

^∗^ *p* < 0.05 compared to corresponding values in healthy controls. ^#^*p* < 0.05 compared to corresponding values in non-cirrhotic PBC patients. PBC: primary biliary cholangitis; BMI: body mass index; GDF15: growth differentiation factor 15; AST: aspartate aminotransferase; ALT: alanine aminotransferase; TBIL: total bilirubin; DBIL: direct bilirubin; GGT: gamma-glutamyl transpeptidase; ALP: alkaline phosphatase; TBA: total bile acids; ALB: albumin; GLB: globulin; INR: international normalized ratio; CRP: C-reactive protein; MELD score: Model for End-stage Liver Disease score.

**Table 2:** Univariate regression analysis of clinical and laboratory parameters associated with GDF15 in cirrhotic PBC patients.

Variables	*B*	SE	*p* value	95% CI	*R* ^2^
TBIL (*μ*mol/L)	0.517	0.108	<0.001	0.290-0.745	0.558
DBIL (*μ*mol/L)	0.435	0.078	<0.001	0.272-0.599	0.635
TBA (*μ*mol/L)	0.522	0.163	0.005	0.179-0.865	0.362
ALB (g/L)	-0.052	0.009	<0.001	-0.072 to -0.032	0.629
CRP (mg/L)	0.455	0.101	<0.001	0.243-0.668	0.547
MELD score	0.577	0.140	0.001	0.283-0.872	0.485
Mayo risk score	1.950	0.460	<0.001	0.983-2.917	0.499

TBIL: total bilirubin; DBIL: direct bilirubin; TBA: total bile acids; ALB: albumin; CRP: C-reactive protein; MELD score: Model for End-stage Liver Disease score; CI: confidence interval.

**Table 3:** Multivariate regression analysis of clinical and laboratory parameters associated with GDF15 in cirrhotic PBC patients.

Variables	*B*	SE	*p* value	95% CI	*R* ^2^
ALB (g/L)	-0.036	0.016	0.038	-0.070 to -0.002	0.671

ALB: albumin; SE: standard error; CI: confidence interval.

**Table 4:** Clinical and laboratory parameters of biochemical responders and non-responders in cirrhotic PBC patients.

Feature	Biochemical responders (*n* = 7)	Biochemical non-responder (*n* = 13)	*p* value
Age (years)	53.29 ± 6.01	54.31 ± 2.66	0.859
Gender (male)	3	4	0.651
GDF15 (pg/mL)	2983.14 ± 757.12	5972.83 ± 809.45	0.014
Weight changes (kg)	−0.67 ± 0.33	−2.00 ± 0.66	0.209
ALT (U/L)	79.29 ± 19.16	89.39 ± 21.59	0.877
AST (U/L)	76.71 ± 12.28	115.77 ± 19.95	0.211
TBIL (*μ*mol/L)	44.16 ± 22.34	123.44 ± 22.87	0.003
DBIL (*μ*mol/L)	31.25 ± 20.68	90.66 ± 17.30	0.008
GGT (U/L)	220.43 ± 73.73	357.62 ± 102.96	0.536
ALP (U/L)	225.71 ± 40.25	293.77 ± 32.23	0.249
TBA (*μ*mol/L)	88.33 ± 27.50	156.35 ± 24.38	0.097
ALB (g/L)	36.80 ± 1.47	32.87 ± 1.23	0.059
GLB (g/L)	35.10 ± 4.10	36.26 ± 2.79	0.819
INR	1.07 ± 0.07	1.40 ± 0.13	0.040
CRP (mg/L)	7.20 ± 3.05	21.12 ± 5.71	0.038
MELD score	7.36 ± 2.67	11.63 ± 1.68	0.135
Mayo risk score	5.23 ± 0.42	7.30 ± 0.35	0.002

PBC: primary biliary cholangitis; GDF15: growth differentiation factor 15; AST: aspartate aminotransferase; ALT: alanine aminotransferase; TBIL: total bilirubin; DBIL: direct bilirubin; GGT: gamma-glutamyl transpeptidase; ALP: alkaline phosphatase; TBA: total bile acids; ALB: albumin; GLB: globulin; INR: international normalized ratio; CRP: C-reactive protein; MELD score: Model for End-stage Liver Disease score.

## Data Availability

The datasets used and/or analysed during the current study are available from the corresponding author upon reasonable request.
